# Integrated quantitative first-pass cardiac perfusion MRI protocol

**DOI:** 10.1186/1532-429X-13-S1-P60

**Published:** 2011-02-02

**Authors:** Elodie Breton, Daniel Kim, Sohae Chung, Leon Axel

**Affiliations:** 1 NYU Langone Medical Center, New York, NY, USA

## Introduction

The proposed contrast-enhanced first-pass perfusion cardiovascular MR (CMR) protocol integrates some recent technical MRI advances towards quantitative analysis of perfusion CMR images: fast multi-slice pulse sequence [1], robust saturation pulse [2], accurate dedicated AIF imaging [3], signal-to-concentration modeling [4], and higher SNR at 3T.

## Purpose

To evaluate an integrated first-pass perfusion cardiovascular MR (CMR) protocol designed to determine absolute contrast-agent concentrations in blood and tissues.

## Methods

**Figure 1:**
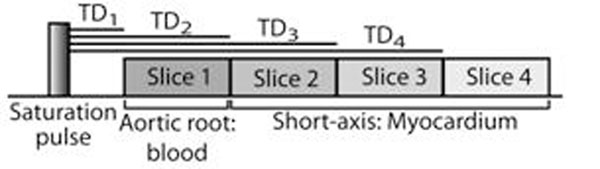
Schematic diagram of the mulit-slice with sequential TD first-pass perfusion CMR pulse sequence

A multi-slice saturation recovery (SR) pulse sequence with sequential SR time delays (TD) after a non-selective saturation pulse [2] was implemented at 3T (Fig.1). The rationale for this acquisition scheme was to acquire a dedicated arterial input function (AIF) image with a short TD (50ms) in the aortic root and short-axis myocardial images with longer TD values (~150-400ms), to allow for the different amounts of T_1_ shortening expected in blood and wall. First-pass perfusion CMR was performed in 7 volunteers (0.05mmol/kg, Gd-DTPA). A signal-to-concentration model was applied to calculate Gd-DTPA concentrations in blood and tissues [4,5]. A proton density-weighted (PDw) image was acquired in the first heartbeat, without the saturation pulse, in order to normalize the image signal, and obtain a theoretical signal-to-T_1_ relationship based on Bloch equation in the center of k-space. Gd-DTPA concentrations were calculated assuming: fast water exchange condition [6], longitudinal relaxivity r_1_=3.8L.mmol^-1^.s^-1^[7], and baseline T_1_ measured with a multi-point SR fit. TurboFLASH imaging parameters included: FOV=350mm×315mm, slice thickness=8mm, matrix=160×144, in-plane resolution=2.2mm×2.2mm, TE/TR=1.2/2.4ms, flip angle 10°, temporal resolution=114ms, tSENSEx3, centric k-space trajectory, and receiver bandwidth=1008Hz/pix. Total image acquisition time was 523ms for the acquisition of 4 slices, namely aortic root and SA base, mid, and apex levels, with respective TD values 50-164-278-393ms. Contours for the blood and left ventricle were drawn manually, and the myocardium was divided into 6 (base-mid) or 4 (apex) standard segments.

## Results

**Figure 2:**
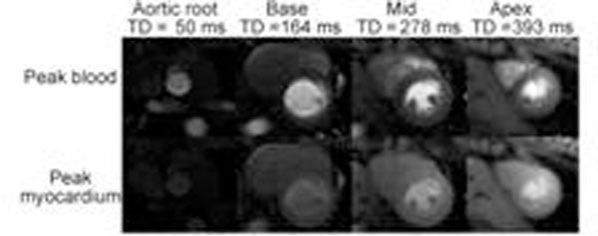
Representative images at peak blood and peak myocardium concentrations.

Representative images at peak contrast in blood and myocardium are shown Fig.2, along with representative AIF and myocardial segment time-responses. The peak blood signal was not clipped in the short TD=50ms AIF images. Normalized signal in the myocardium increased along with TD; however similar [Gd-DTPA] were measured in all 3 short-axis images. First-pass perfusion peak Gd-DTPA concentrations were 3.95±0.080, 0.26±0.07mM in the blood and myocardium, respectively.

**Figure 3:**
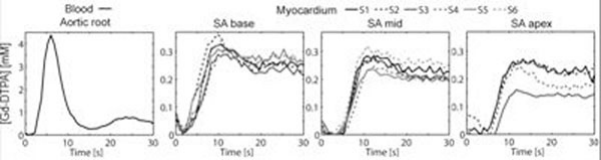
Representative [Gd-DTPA] times-curves in blood in the aortic root and in 6 cardiac segments in cardiac short-axis images.

## Conclusions

The proposed integrated first-pass perfusion CMR protocol at 3T produced AIF and myocardial wall Gd-DTPA concentrations consistent with previously published results. Future work includes evaluation of the integrated protocol in cardiac patients.
